# Prevalence of Total Physical Activity, Muscle-Strengthening Activities, and Excessive TV Viewing among Older Adults; and Their Association with Sociodemographic Factors

**DOI:** 10.3390/ijerph15112499

**Published:** 2018-11-08

**Authors:** Chien-Yu Lin, Jong-Hwan Park, Ming-Chun Hsueh, Wen-Jung Sun, Yung Liao

**Affiliations:** 1 Institute of Health Behaviors and Community Sciences, College of Public Health, National Taiwan University, Taipei 100, Taiwan; chienyulin@ntu.edu.tw; 2 Health Behaviors & Disease Prevention Research Group, Institute of Convergence Bio-Health, Dong-A University, Busan 49201, Korea; jpark@dau.ac.kr; 3 Department of Physical Education, National Taiwan Normal University, Taipei 106, Taiwan; boxeo@ntnu.edu.tw; 4 Family Medicine Division, Taipei City Hospital Zhongxing Branch, Taipei 103, Taiwan; 5 Center of R/D in Community Based Palliative Care, Taipei City Hospital, Taipei 103, Taiwan; 6 Department of Health Promotion and Health Education, National Taiwan Normal University, Taipei 106, Taiwan

**Keywords:** behavioral epidemiology, guidelines, physical activity, sedentary behavior

## Abstract

The study aimed to describe the prevalence of meeting moderate-to-vigorous-intensity physical activity (MVPA), muscle-strengthening (MS) activities, and television (TV) viewing guidelines, and their association with sociodemographic factors. Data from older adults aged 65 or above were sampled by age and sex to the population aged 65+ years for each area in Taiwan and collected through telephone interviews. The prevalence of meeting MVPA and MS activities, MVPA and MS activities guidelines, and excessive TV viewing were calculated. We also investigated their associations with sociodemographic variables using logistic regression analyses. A total of 1068 older adults (response rate: 32.5%) participated in the present study. 79.4% met the MVPA guidelines (150 min weekly), 25.3% met the MS guidelines (twice a week), 22.4% met both MVPA and MS guidelines, and 53.1% engaged in excessive TV viewing (more than or equal to two hours per day). Overall, in old age, low educational level was associated with lower odds of meeting MVPA and MS activities, and both the MVPA and MS activity guidelines; while living alone and having no full-time job had higher odds of excessive TV viewing. A large number of older adults do not meet the MS recommendations, but are engaged in excessive TV viewing. Our findings may be important for public health interventions to promote MS and avoid excessive TV viewing, especially for at-risk subgroups.

## 1. Introduction

Physical inactivity is a leading cause of decreasing life expectancy and premature mortality [1,2]. For the prevention of chronic diseases and disability, it is suggested that physical activity become one of the highest priorities for older adults [3]. It is recommended that older adults engage in at least 150 min of moderate-intensity aerobic physical activity weekly, and muscle-strengthening (MS) activities twice a week [4]. However, previous epidemiological studies have mainly focused on moderate-to-vigorous-intensity aerobic physical activity (MVPA) [5,6], but paid relatively less attention to MS activities. To better understand the physical activity levels among older adults, there is an urgent need for research that further defines the descriptive epidemiology of MS activities and the combination of MS activities and aerobic physical activity.

Independent of physical activity, sedentary behavior (i.e., low energy-expenditure activity while in a sitting or reclining posture in waking hours) has become a novel behavioral risk factor among older adults [7]. Greater sedentary time was found to be associated with an increased risk of all-cause mortality, metabolic syndrome, larger waist circumference, and overweightness and obesity in older adults [8]. Among varied types of sedentary behaviors, television (TV) viewing is the most predominant leisure-time sedentary behavior among older adults [9]. A recent study also reported that even high physical-activity levels (i.e., 60–75 min per day) cannot eliminate the risks posed by excessive TV viewing [10]. Considering the deleterious health impact of TV viewing, in addition to MS activities and aerobic physical activity, it is also crucial to describe the epidemiology of excessive TV viewing and its sociodemographic characteristics in older adults.

Given that promoting aerobic physical and MS activities and reducing TV viewing time is the priority among older adults [3], the aim of this study was to investigate the prevalence of adherence to MVPA and muscle-strengthening activities, both MVPA and MS activities, and excessive TV viewing among a population-representative sample of older adults aged 65 or above, and their sociodemographic correlations.

## 2. Materials and Methods

### 2.1. Participants

This study collected data using the Computer Assisted Telephone Interview system. We conducted a random-digit dialing telephone-based survey based on the White Pages in Taiwan. Individuals with a household telephone comprised more than 90% of the population [11]. Respondents aged 65 or above were selected using a stratified random-sampling process. We categorized the respondents based on the counties/cities where they reside at the first stage and divided the 22 counties/cities into four areas. We used area division as the geographic unit that was an important consideration when making decisions in Taiwan’s policy [12]. In the second stage, we stratified them by age (65–74, 75–84, 85+ years) and sex. The number of respondents who aged 65+ years sampled by age and sex for each area according to the proportion to the total population aged 65+ years in Taiwan. The person from the household who was aged 65+ years was selected as the respondent. While there was more than one person aged 65+ years, we only randomly selected one person to complete the survey.

A total of 9844 telephones were contacted. Apart from eligible respondents, the majority of telephones had no older adults living in the household (28.1%), not answering the phone (24.1%), phone number no longer valid (11.6%), and other reasons (2.9%). Among 3282 respondents with respondence, some older adults were not included in our analyses because of refusing to be interviewed (61.2%), lack of the ability to be interviewed (e.g., physical and mental illness) (4.9%), and other reasons (1.4%). There were 1068 respondents (mean age = 73 years, standard deviation = 6.1) who completed the survey (response rate: 32.5%). To increase the response rate, each interview was controlled in 15–20 min. Respondents were not offered any rewards, and each of them was asked for their verbal consent before the beginning of their telephone interview. The study protocols were approved by the Research Ethics Committee (REC) of National Taiwan Normal University (REC number: 201706HM020).

### 2.2. Measurements

For the present study, we collected data on physical activity, MS activities, TV viewing, and sociodemographic characteristics.

#### 2.2.1. Total Physical Activity

To assess participation in physical activities, we used the Taiwanese version of the International Physical Activity Questionnaire-long version (IPAQ-LV). IPAQ has been previously validated among middle-aged to older adults [13,14]. The questionnaire has been shown to have adequate reliability and validity when assessed against accelerometers [15]. Participants reported frequency and estimated the time they spent walking, in moderate-intensity activities (e.g., hiking, going downstairs, and baseball), and vigorous physical activities (e.g., running, going upstairs, and basketball). We calculated the total time spent in MVPA and divided it into ‘sufficient MVPA’ (more than or equal to 150 min per week) and ‘insufficient MVPA’ (less than 150 min per week), according to the recommendation for older adults [6].

#### 2.2.2. Muscle-Strengthening Activities

Those who reported engagement in MS activities were asked whether they had done any MS activities in the previous week other than MVPA. If they answered affirmatively, they were further asked: “How many times did you do MS activities last week?” Notably, the questions covered the MS activities that participants had already mentioned in their responses to questions about MVPA, and potentially any other MS activities that are not a part of MVPA. Similar questions have previously been used in studies in Japan [16]. According to the World Health Organization [6], and consistent with past studies [16,17,18], regular MS activity was considered as two days or more per week.

#### 2.2.3. TV Viewing

To determine the time spent watching TV or videos, we referred to the questionnaire which was used to assess older adults’ sedentary behavior in previous studies [19]. The item of TV viewing has been translated into Chinese and has shown acceptable test–retest reliability (ρ = 0.52) and validity (ρ = 0.30) [14]. There are a series of questions on such sedentary activities as TV viewing, computer use, and reading. Among these types of sedentary behaviors, TV viewing had confirmed test–retest reliability (ρ = 0.78) [19]. We dichotomized the TV viewing time based on a previous study, which reported that spending more than two hours per day on TV viewing was associated with health risks [20]. Therefore, we divided TV viewing into “excessive TV viewing (more than two hours per day)” and “low TV viewing (less than two hours per day)”.

#### 2.2.4. Sociodemographic Characteristics

Participants were asked to self-report on sociodemographic characteristics, including sex, age, educational level, marital status, living status, and occupational status. Age was categorized into “65–74 years”, “75–84 years”, and “85+ years”. Educational level was categorized into “lower than university degree (low)” and “university degree or higher (high)”. Marital status was categorized into “married” and “unmarried” (including single, divorced, and widowed). Living status was dichotomized was categorized into “living with others” and “living alone”, and occupational status was categorized into “no full-time job” and “full-time job”.

### 2.3. Statistical Analysis

The present study calculated percentages of four categories as follows: (i) meeting the MVPA guidelines; (ii) meeting the MS activity guidelines; (iii) meeting both MVPA and MS activity guidelines; and (iv) excessive TV viewing. Pearson’s chi-square tests were used to detect differences between the proportions of the selected variables. A series of multiple logistic regression analyses was conducted to examine the associations of MVPA, muscle-strengthening activity, and TV viewing with sociodemographic characteristics. We reported adjusted odds ratios (OR) with corresponding 95% confidence intervals (95% CI). Statistical analyses were performed using IBM SPSS 23.0 software (IBM, Armonk, NY, USA).

## 3. Results

### 3.1. Participants’ Sociodemographic Characteristics

Data were available for 1068 older adults aged 65 or above. As shown in Table 1, in the total sample, 79.4% of participants met the MVPA guidelines, 25.4% of the participants met the muscle-strengthening activity guidelines, 22.5% of the participants met both MVPA and muscle-strengthening activity guidelines, and 53.1% of the participants engaged in excessive TV viewing. Notably, among these older adults who did not meet the MS activity guidelines, only 0.3% of our respondents (N = 3) reported engaging MS activity once a week, whereas others reported no MS activity at all (data were not shown in Table 1). Chi-square test analysis revealed proportional differences between sociodemographic characteristics and various guideline variables.

### 3.2. Association between Sociodemographic Characteristics and Various Guidelines

Table 2 shows logistic regression analyses after full adjustment for socioeconomic characteristics. There were different patterns within meeting MVPA, muscle-strengthening activity, and both MVPA and muscle-strengthening activity guidelines; and excessive TV viewing. Regarding sociodemographic characteristics and the category of meeting MVPA guidelines, compared to older adults aged 65–74 years, those aged 85+ years were less likely to meet the MVPA (adjusted OR = 0.46, 95% CI = 0.26–0.81), muscle-strengthening activity (adjusted OR = 0.37, 95% CI = 0.17–0.80), and both MVPA and muscle-strengthening activity guidelines (adjusted OR = 0.43, 95% CI = 0.20–0.94). Further, older adults with a lower educational level were less likely to meet the MS activity guidelines (adjusted OR = 0.64, 95% CI = 0.48–0.86), and both MVPA and MS activity guidelines (adjusted OR = 0.64, 95% CI = 0.47–0.87). Older adults who lived alone were more likely to engage in excessive TV viewing compared to those who lived with others (adjusted OR = 1.57, 95% CI = 1.03–2.38). Finally, older adults without full-time jobs were more likely to meet both MVPA and MS activity guidelines (adjusted OR = 2.40, 95% CI = 1.31–4.42) as well as engage in excessive TV viewing (adjusted OR = 1.62, 95% CI = 1.07–2.45).

## 4. Discussion

This study is the first to concurrently examine the prevalence and sociodemographic correlations of meeting MVPA, MS activity, and sedentary-behavior guidelines among Taiwanese older adults. The most important finding of this study was that majority (77.5%) of Taiwanese older adults did not meet the full physical activity recommendations that incorporate both MVPA and MS activity, although 79.4% of these older adults met the MVPA guidelines alone. These findings may have two significant implications for informing policy makers and intervention designers. First, in addition to promoting aerobic types of physical activity among older adults, there is an urgent need to increase awareness of the importance of muscle-strengthening activities, and design comprehensive approaches to promote and support both aspects of physical activity (aerobic and MS) among Taiwanese older adults. Second, future physical-activity epidemiological studies should investigate both aspects of physical activity to avoid underestimation of the true situation of physical inactivity among older adults.

The present study showed that 79.4% of older Taiwanese adults met the MVPA guidelines, which was higher than a previous Taiwanese study that reported 39.4% of older adults engaged in sufficient leisure-time physical activity [14]. The disparity may be because we measured the comprehensive physical activity (i.e., household, transport, work, and leisure time) rather than only a specific-context leisure-time physical activity. Comparing the prevalence of physical activity from older adults from Western countries to those in Taiwan, we found that of Taiwanese older adults to also obviously be higher than the older adults in the U.S. (27.3%–44.3%) [21] and New Zealand (46.2%) [22]. Moreover, in the present study, our findings showed that more than half of the older adults (53.1%) engaged in excessive TV viewing (two hours or more per day), which was slightly higher than another finding (44.3%) among Japanese older adults [23]. These findings showed that in addition to physical activity, there is also an urgent need for effective strategies of reducing the TV viewing time among older adults.

Consistent with previous studies, individuals who were older were less likely to meet MVPA and strength-training exercise alone and together compared to their younger counterparts [24]. One possible explanation is that the decline in physical activity with age, including muscle-strengthening activity, could contribute to a decline in physical mobility. Furthermore, older adults might fear injuries, making them less likely to engage in physical activity or muscle-strengthening activity [25]. Our findings also showed that adults who had a lower educational level were less likely to meet both MVPA and muscle-strengthening activity guidelines. One possible reason for this might be the finding that lower-educated individuals have limited health consciousness and less knowledge about the advantages of physical activity compared to higher-educated individuals [24]. Moreover, older adults living alone had a 57% greater probability of watching TV compared to two hours of TV viewing in a day among adults living with other people. Previous studies showed that TV viewing could provide older adults a means of isolation reduction, communication substitution, and social companionship [26]. Therefore, watching TV might be a common leisure-time activity for older adults living alone to mitigate loneliness. Notably, older adults who did not have full-time jobs were more likely to meet both MVPA and MS activity guidelines as well as excessively watch TV. Older adults without a full-time job had more flexible time to engage in some active and sedentary leisure activities. Therefore, how to encourage these older adults to spend more time on active behavior, but not sedentary behavior, needed further interventions. Findings have implications on identifying specific determinants of high-risk populations and public health interventions.

There were some limitations that need to be taken into consideration in our study. First, the cross-sectional design could not determine causality. Second, we used self-reported measurement to obtain the information on MVPA, muscle-strengthening activity, and sedentary behavior, and this might somewhat result in bias [27]. To improve the validity of measurements, we suggest that further studies be conducted using both objective and subjective measures to assess time spent in physical activities and sedentary behaviors. Third, dichotomized data may limit the interpretation of the association of meeting the recommendation of MVPA and MS activity, and TV viewing with sociodemographic characteristics. For example, the item of job status was only measured by “full-time job” and “not full-time job” in our study. However, the category of “not full-time job” may contain both “no job” and “part-time job”. Further detailed information is needed in future studies. Finally, compared to the general population, our data were more male, younger, highly educated, and more married. The study had a limited representative population sample because it depended on a telephonic survey based on residential telephone numbers. Therefore, sections of the population without a household telephone (approximately 7.3% of entire population in 2016) [11] and living in care institutions (approximately 1.5% of entire population aged 65+ years in 2016) [28] were unreachable in this study. Therefore, the findings may not be generalizable to the entire population of Taiwan.

## 5. Conclusions

There were a large number of older adults did not meet MS recommendations but engage in excessive TV viewing time. These findings provided epidemiological evidence in the context of Asian countries and identified specific high-risk subgroups.

## Figures and Tables

**Table 1:** Prevalence of meeting moderate to vigorous-intensity physical activity (MVPA) guidelines, muscle-strengthening (MS) activity guidelines ^b^, combined MVPA–MS activity guidelines ^c^, and engaging in excessive TV viewing ^d^ by sociodemographic factors.

	Met MVPA Guidelines ^a^		Met Muscle-Strengthening Activity Guidelines ^b^		Met Both MVPA and Muscle-Strengthening Activity Guidelines ^c^		Excessive TV Viewing ^d^	
	*n*	%	*n*	%	*n*	%	*n*	%
Total	848	(79.4%)	271	(25.4%)	240	(22.5%)	567	(53.1%)
**Sex**								
Male	424	(50.0%)	145	(53.5%)	129	(53.8%)	266	(46.9%)
Female	424	(50.0%)	126	(46.5%)	111	(46.2%)	301	(53.1%)
*p*-value	0.631		0.233		0.235		0.016 *	
**Age**								
65–74 years	554	(65.3%)	186	(68.6%)	164	(68.3%)	360	(63.5%)
75–84 years	253	(29.8%)	77	(28.4%)	68	(28.3%)	178	(31.4%)
85+ years	41	(4.8%)	8	(3.0%)	8	(3.3%)	29	(5.1%)
*p*-value	0.007 *		0.034 *		0.103		0.218	
**Education level**								
High	264	(31.1%)	104	(38.4%)	94	(39.2%)	161	(28.4%)
Low	584	(68.9%)	167	(61.6%)	146	(60.8%)	406	(71.6%)
*p*-value	0.132		0.001 *		<0.001 *		0.208	
**Marital status**								
Married	648	(76.4%)	214	(79.0%)	192	(80.0%)	416	(73.4%)
Unmarried	200	(23.6%)	57	(21.0%)	48	(20.0%)	151	(26.6%)
*p*-value	0.319		0.153		0.081		0.053	
**Living status**								
Living with others	729	(86.0%)	233	(86.0%)	205	(85.4%)	472	(83.2%)
Living alone	119	(14.0%)	38	(14.0%)	35	(14.6%)	95	(16.8%)
*p*-value	0.846		0.949		0.822		0.009 *	
**Occupational status**								
Full-time job	89	(10.5%)	20	(7.4%)	13	(5.4%)	45	(7.9%)
Not full-time job	759	(89.5%)	251	(92.6%)	227	(94.6%)	522	(92.1%)
*p*-value	0.540		0.075 *		0.005 *		0.009 *	

^a^ Prevalence of respondents who reported engaging in at least 150 min of moderate-intensity physical activity per week or 75 min of vigorous-intensity physical activity per week, or an equivalent combination of both. ^b^ Prevalence of respondents who reported participating in muscle-strengthening physical activity at least two times per week. ^c^ Meeting both the MVPA and strength-training guidelines. ^d^ To be classified as ‘Excessive TV Viewing’, respondents had to report more than 2 h per day spent in TV viewing; * *p* < 0.05.

**Table 2:** Association between sociodemographic characteristics and meeting MVPA guidelines, meeting MS activity guidelines, meeting the combined MVPA–MS activity guidelines, and engaging in excessive TV viewing.

Sociodemographic Characteristics	Met MVPA Guidelines ^a^		Met Muscle-Strengthening Activity Guidelines ^b^		Met Both MVPA and Muscle-Strengthening Activity Guidelines ^c^		Excessive TV Viewing ^d^	
	Adjusted OR	(95% CI)	Adjusted OR	(95% CI)	Adjusted OR	(95% CI)	Adjusted OR	(95% CI)
**Sex (ref: male)**								
Female	1.08	(0.79, 1.47)	0.85	(0.64, 1.13)	0.85	(0.63, 1.14)	1.25	(0.98, 1.61)
Age group (ref: 65–74 years)								
75–84 years	1.01	(0.72, 1.41)	0.87	(0.64, 1.19)	0.87	(0.63, 1.21)	1.10	(0.84, 1.45)
85+ years	0.46	(0.26, 0.81)	0.37	(0.17, 0.80)	0.43	(0.20, 0.94)	0.67	(0.39, 1.14)
Education level (ref: high)								
Low	0.77	(0.55, 1.08)	0.64	(0.48, 0.86)	0.64	(0.47, 0.87)	1.18	(0.90, 1.54)
Marital status (ref: married)								
Unmarried	0.88	(0.58, 1.32)	0.82	(0.54, 1.22)	0.70	(0.45, 1.07)	1.05	(0.75, 1.48)
Living status (ref: living with others)								
Living alone	1.15	(0.69, 1.89)	1.18	(0.74, 1.91)	1.35	(0.82, 2.22)	1.57	(1.03, 2.38)
Occupational status (ref: full-time job)								
Not full-time job	0.87	(0.51, 1.46)	1.65	(0.99, 2.77)	2.40	(1.31, 4.42)	1.62	(1.07, 2.45)

^a^ Prevalence of respondents who reported engaging in at least 150 min of moderate-intensity physical activity per week or 75 min of vigorous-intensity physical activity per week, or an equivalent combination of both. ^b^ Prevalence of respondents who reported participating in muscle-strengthening physical activity at least two times per week. ^c^ Meeting both the MVPA and strength training guidelines. ^d^ To be classified as ‘Excessive TV Viewing’, respondents had to report more than 2 h per day spent in TV viewing. Abbreviation ref: reference group.
